# Self-reported and the actual involvement of community pharmacy professionals in the management of acute childhood diarrhea in Jazan Province, Saudi Arabia: Simulated patient visits and survey study

**DOI:** 10.1016/j.jsps.2022.07.012

**Published:** 2022-07-29

**Authors:** Eman Merghani Ali, Saad S. Alqahtani, Nabeel Kashan Syed, Otilia J.F. Banji, Yahia Ali Mashragi, Mohammed Ali Ageel, Bashaer Abdullah Hakami, Majed Hassan Mathkur, Renad Mehsen Solan, Amani Khardali, Rina Tripathi

**Affiliations:** a Department of Pharmacy Practice, College of Pharmacy, Jazan University, Jazan, Saudi Arabia; b Pharmacy Practice Research Unit, College of Pharmacy, Jazan University, Jazan, Saudi Arabia; c Pharmacy Department, King Fahad Hospital, Jazan, Saudi Arabia; d Pharmacy Department, Armed Force Hospital, Jazan, Saudi Arabia

**Keywords:** Community pharmacy professionals, Simulated patient visits, Self-report survey, Knowledge, Attitudes, Practices, Acute childhood diarrhea, Saudi Arabia

## Abstract

**Background:**

Acute childhood diarrhea is one of the most common causes of dehydration, and if severe, can potentially lead to death as well. The present study was aimed at evaluating the knowledge and attitudes of community pharmacy professionals towards the management of acute childhood diarrhea and comparing them with their actual practices in Jazan Province, Kingdom of Saudi Arabia (K.S.A).

**Methods:**

Simulated patient visits and a cross-sectional survey making use of a 27-item self-report questionnaire were conducted amongst a sample of 303 community pharmacy professionals (51.2 % male and 48.8 % female) with an age range of 25–56 years, between August 1 and December 1, 2021, in Jazan Province, Saudi Arabia. Convenience sampling technique was used for the recruitment of the participants.

**Results:**

Significant positive correlations were seen between knowledge score (self-report survey) and practice score (simulated patient visit) regarding patients’ history taking (r = 0.65; *p < 0.01*), drug recommendations (r = 0.71; *p < 0.01*) and providing information regarding food and fluid intake (r = 0.44; *p < 0.01*)*.* The alpha coefficients for all the items of the survey instrument were noted to be greater than (or) equal to the 0.70 threshold for almost all sections, hence indicating good reliability and internal consistency of the developed scale.

**Conclusion:**

In the present study, even though we observed improved participants’ performance during the self report survey, their performance level greatly dropped in actual practice. This warrants for a need for educational programs to improve their actual dispensing practices. The present study has also shown simulated patient visits to be a reliable, simple and a robust method of assessing the actual dispensing practices of community pharmacy professionals.

## 1 Introduction

Acute diarrhea is defined as the passing of loose stools lasting <14 days (Thapar & Sanderson, 2004). In comparison, persistent symptoms for >14 days are referred to as persistent diarrhea, while continual symptoms in excess of 30 days are referred to as chronic diarrhea (Thapar & Sanderson, 2004). Globally, acute diarrhea is the second leading cause of death in children below the age of 5 years, killing around 525,000 children every year. **(**Abegaz et al., 2016, World Health Organization, 2017**)**. There are nearly 1.7 billion cases of childhood diarrheal disease every year globally (WHO, 2017**)**. Dehydration caused by diarrhea accounts for approximately 1.5 to 2.5 million deaths/year globally (WHO, 2017). In diarrhea, altered bowel movements reduce the consistency of the fecal discharge and hence it leads to the fecus containing more water in the stools than normal and in some cases can also contain blood (Diwan et al., 2015). Serious diarrhea associated complications include malnutrition and in the most severe cases could be death due to the depletion of salts and water from the body (Diwan et al., 2015). The clinical manifestations include sudden onset of nausea, vomiting, abdominal cramps, headache, and malaise. The management strategies focus on the prevention, management of dehydration, followed by the administration of zinc supplements. As a home remedy, dehydration can be prevented by consuming plenty of fluids orally which needs to be initiated early, at the onset of diarrheal symptoms (Ogbo et al., 2014). Food-based fluids like soup, rice and water are also recommended, along with the intake of twice the normal quantity of water. Management for dehydration is possible at community health care centers, and the best management strategy that which is commonly followed is the administration of oral rehydration salt (ORS). Zinc supplementation has also been reported to be effective in declining the duration, incidence, and severity of diarrheal episodes. (Santosham et al., 1997). As part of its management, feeding is also contemplated, incorporating micro-nutrients that which are easily digestible, thereby preventing weight loss in the affected child.

On the other hand, a comprehensive and an effective management regimen has not been fully developed to overcome this problem. Moreover, the use of antibiotics may not serve as a magic bullet for all microorganisms. In turn, their long-term use can lead to further deterioration owing to their side effects as well as leading to antibiotic resistance (Hlashwayo et al., 2021). The use of adsorbents like kaolin, pectin, and activated charcoal may be slightly beneficial as they change the consistency of the stool but, unfortunately they do not restore the fluids and salt already lost (Mengistu et al., 2019). In addition to this, anti-motility agents such as tincture of opium or loperamide have deleterious consequences when given to children less than five years of age (Turck et al., 1999). Keeping this in mind, symptomatic management might be a more effective and a more favorable option. For most children with infectious diarrheal illnesses, pharmacotherapy is not advisable; however, the use of ORS and food additives might be extremely beneficial (Karim et al., 2004). As diarrhea is considered a significant public health problem, many countries are facing challenges in dealing with it. Some of the contributing factors could be improper personal hygiene, lack of knowledge, and misguided management (WHO, 1994, WHO, 1994). It is the responsibility of the pharmacists to create awareness programs, respond to health-related queries, guide the general public, and ultimately work towards the goal of minimizing the overall mortality associated with childhood diarrhea. Community pharmacists can also contribute in this regard by initiating a meticulous approach towards the diagnosis and management of diarrhea and by providing proper referral to medical care when needed. As health care professionals, community pharmacists should be involved in the management as well as in the prevention of acute diarrhea and should make appropriate interventions as and when needed in its management protocol. The community pharmacists are the most easily accessible and the most approachable health care professionals (Mengistu et al., 2019) from the public point of view and hence can participate in the management of an array of healthcare issues and they can also help in responding to explicit health-related queries (Abbaher & Elnima, 2013). They constantly get involved in resolving public health issues due to their proximity to the community as well as due to their constant exposure to similar cases in the community. The role of community pharmacists in the management of minor illnesses is gaining wide-spread recognition and acceptance. Worldwide, the number of medicines that which are available to be purchased without prescription are increasing (Watson et al., 2004). Community pharmacy professionals can play an extremely impactful role in guiding the general public in terms of diagnosis, prevention, and management of acute childhood diarrhea as well.

In Jazan Province, located in the southwestern region of Saudi Arabia, community pharmacy professionals take the responsibility of prescribing and dispensing over the counter (OTC) medications like ORS as a part of the management and prevention of dehydration associated with acute childhood diarrhea (Mengistu et al., 2019). Prescription only medications like the anti-microbials cannot be prescribed by the community pharmacy professionals without the proper identification of the causative organism and incase of suspected infections are required to refer the patients for further diagnosis (Mengistu et al., 2019). Inspite of the growing involvement of the community pharmacy professionals in the management of a wide-array of health care issues, there are persisting concerns as to whether the community pharmacists as well as their staff provide appropriate advice regarding the use of OTC medicines to their patients (Watson et al., 2004). There have been instances wherein the pharmacists themselves have expressed a pressing need for educational initiatives (Scottish Department Executive, 1992). There are major hindrances in identifying as well as following up patients in community pharmacy settings (Sinclair et al., 1995), some of which include the potential for selection bias and the Hawthorne effect (i.e. a change in behavior as a response to being observed and assessed) (Sedgwick and Greenwood, 2015, Watson et al., 2004). In order to identify and address these issues, covert simulated patients have been successfully used in pharmacy practice research. Simulated patients (also referred to as standardised clients (or) mystery shoppers) are trained individuals who present particular scenarios (e.g., mother of a child suffering from acute diarrhea) and are often indistinguishable from actual patients and who visit different pharmacies in order to assess the different aspects of customer care being provided by the pharmacy staff (Collins et al., 2021, Watson et al., 2004, Watson et al., 2009). Simulated patient visit is a simple method that which provides an effective way of documenting clients' real time, unconscious behaviors and their practices (Mengistu et al., 2019). It is now widely being used worldwide to assess the dispensing practices of the community pharmacists as well (Diwan et al., 2015). Hence, in the present study a self-report survey as well as simulated patient visits were used to assess the involvement of community pharmacy professionals in the management of acute childhood diarrhea in Jazan Province, Saudi Arabia. The knowledge and the attitudes of community pharmacy professionals were assessed using a validated, structured self-reported questionnaire whereas their corresponding practices were assessed using simulated patient visits.

## 2 Methods

### 2.1 Study design and study population

Cross-sectional study using a validated 27 item-self-report survey as well as simulated patient visits was used to collect data amongst a sample of 303 community pharmacy professionals.

### 2.2 Inclusion and exclusion criteria

For the participants to be included in the survey, they were required to (i) be working in a community pharmacy (registered pharmacists and pharmacy technicians) (ii) be at least 26 years of age (iii) have an understanding of English (or) Arabic languages (iv) be willing to provide their written consent and (v) be willing to participate in the study by answering the questionnaire. However, in simulated patient visits a convenient sample of a few community pharmacy professionals interacting with simulated cases was included. Those participants not meeting the inclusion criteria were excluded from the study.

### 2.3 Data collection tools

Data was collected by making use of a validated (face and content validation), structured, and pre-coded self-report questionnaire printed as hard copies (Ahsan et al., 2021). The survey comprised of 2 sections. Section one consisted of 5 questions related to participants’ demographics. Section 2 had 4 sub-sections comprising of 22 questions relating to: participants’ knowledge towards history taking (9 items), knowledge of drug recommendations (5 items), attitudes towards information on food and fluid intake (5 items), and practices (3 items) towards the preparation and storage of ORS. The self-report survey was developed by the authors after a thorough and careful deliberation wherein similar literature was reviewed. Some of the items in the survey were adapted from Mengistu et al. (2019). Based on these deliberations a final version of the questionnaire was prepared and approved comprising of 27 items (*see* Appendix 1).

### 2.4 Measures

#### 2.4.1 Demographics

Participants of the present study reported their gender, age group (in years), exact age, qualification, job profile and their experience (in years). These were used as explanatory variables (Ahsan et al., 2021).

The participants also recorded their responses to questions assessing their knowledge towards history taking of patients’ with acute childhood diarrhea. An item example includes, “*Patients’ age is an important component in the history taking of the patients’ suffering from acute childhood diarrhea?”.* The items of this section have binary responses, from 0 (no) to 1 (yes). The participants also recorded their responses to questions assessing their knowledge towards drug recommendations in acute childhood diarrhea. An item example includes, *“ORS and Zinc supplements are given to alleviate diarrheal symptoms?”.* The items of this section also have binary responses, from 0 (no) to 1 (yes). The participants also recorded their responses to questions assessing their attitude towards information on food and fluid intake. An item example includes, *“Advice to continue normal feeding and increasing the fluid intake during diarrhea?”.* The items of this section were scored on a four point likert scale, from 1 (*strongly disagree*) to 4 (*strongly agree*). The participants also recorded their responses to questions assessing their practices towards preparation and storage of ORS, an item example of this section includes *““Do you know how to prepare ORS?”.* The items of this section also had binary responses, from 0 (no) to 1 (yes).

### 2.5 Development, validation and translation of the study questionnaire

All the items in the study questionnaire were translated into Arabic using the forward–backward translation method (Beaton et al., 2000, Syed et al., 2022). An independent bilingual translator with proficiency in English and Arabic languages was selected to translate the English version into Arabic language. This version was then further reviewed by a study author with bilingual proficiency. Any mistakes if present were discussed with the independent translator and were subsequently resolved after which the final version of the questionnaire was prepared. This final version of the Arabic questionnaire was then back translated into English language by an author also having bilingual proficiency and who was until now kept unaware of the original English version. The forward and backward translated version of the study questionnaires were then reviewed by all the study authors and a final version was prepared and approved (Alnohair et al., 2021, Syed et al., 2022). The approved final version was then piloted in a focus group of 10 % of the sample size for assessing the ease of use and completion time (Syed et al., 2022, Syed et al., 2020). A completion time of around six minutes was noted in the pilot study and all the respondents were able to understand the questions with relative ease. The study questionnaire was validated through face and content validation. The sample of the pilot study was also used to test the reliability and the internal consistency of the instrument and this sample was not included in the final analysis.

### 2.6 Data collection

#### 2.6.1 For self-report survey

Data was collected using a validated, structured, and pre-coded self-report questionnaire (Ahsan et al., 2021). The questionnaire was prepared in English as well as in Arabic languages. The participants selected the version they felt comfortable with. Participants’ informed consent was taken prior to data collection. The inability of the respondents to complete any question (or) any of the sections of the survey rendered the response as incomplete, which were consequently excluded from the final analysis (Ahsan et al., 2021).

#### 2.6.2 For simulated patient visits

In the simulated patient visits, three Pharm.D interns delegating the role of the mother of the afflicted child visited community pharmacies and were hence considered the simulated cases. They had prior training from a skilled and experienced clinical pharmacist on acute childhood diarrhea. These simulated cases were properly trained and were thorough in their approach towards seeking the management and during the process, collecting all the information needed before leaving the community pharmacy and then transcribing the acquired information on data collection forms. Additionally, WHO as well as Diarrhea Disease Control Program guidelines were followed in assessing patients’ treatment along with the instructions provided on food and fluid intake (Ogbo et al., 2014). A total of 100 simulation visits were conducted. Convenience sampling technique was used for participants’ recruitment.

### 2.7 Scoring of the questionnaire

The survey instrument consists of 4 subsections covering three domains (i.e. knowledge, attitude and practices). A total score was calculated for each sub-section by adding the scores of each item. After a thorough and extensive literature review it was decided to use Bloom’s cut off point (Bloom et. al., 1956), according to which any score <60 % of the total score was deemed as poor, where as a score in the range of 60–80 % was categorized as being moderate and, any score >80 % of the total score was deemed to be excellent. (Bloom et al., 1956, Elshammaa et al., 2022, Koo et al., 2015).

### 2.8 Sample size calculation

The sample size was calculated as 285 by using *Raosoft* sample size calculator, based on 95 % confidence interval, 5 % margin of error, an approximate population size of around 1100 community pharmacy professionals along with a 50 % response distribution (Alnohair et al., 2021, Elnaem et al., 2021, Syed et al., 2020, Raosoft, 2020).

### 2.9 Ethical considerations

The protocol, study procedures along with the informed consent form (ICF) were submitted to the Institutional Research Review and Ethics Committee of Jazan University, Jazan Province, K.S.A., prior to the start of the study for getting the required ethical approval. This study was performed in accordance with the principles of the Helsinki Declaration for studies involving human subjects (Approval Number: 1863/2910/1442). Written consent was taken prior to data collection only in the self-report survey study. No consent was taken during the simulated patient visits because taking a written/verbal consent would remove the cover of the simulated patients thereby introducing the potential of “Hawthorne Effect”.

### 2.10 Data analysis

Statistical Package for the Social Science (SPSS Inc., Chicago, IL., USA) statistical software (version 23) was used for data analysis. Upon the completion of the survey, data from the filled questionnaires and the simulated patient visits was scrutinized. The information was cross-checked and subsequently entered into an excel sheet and was later transferred into a SPSS file. The result of the demographics section was expressed in terms of frequency and total percentage. To look for statistically significant associations between the variables Pearson’s Chi-square test was used. For variables with a count <5 Fisher’s Exact test was used. Pearson’s correlation coefficient was used to look for statistically significant associations between continuous variables (i.e. scores of different scales). For determining statistical significance the alpha level was set at 0.05. Shapiro-Wilk and Kolmogorov-Smirnov test were used as part of the preliminary analysis to confirm the normality of data (Syed et al., 2022).

## 3 Results

### 3.1 Socio-demographics

Table 2, depicts the socio-demographic characteristics of the participants. It was noted that percentage of male (n = 148; 48.8 %) and female participants (n = 155; 51.2 %) were relatively similar. The highest percentage of the study sample was observed to be in the age group of 20–30 years (n = 123; 40.6 %). The participants’ mean age was 34.59 years (SD ± 8.07). More than four-fifths of participants (n = 252; 83.2 %) had Pharm.D qualification. It was also noted that more than half of participants (n = 203; 67 %) were working as staff pharmacists. Similarly high percentage of the study sample (n = 188; 62 %) had experience in the range of 5–10 years.

**Table 1:** Simulated patient scenario (Ogbo et al., 2014).

*Anticipated Questions*	*Prepared Answers*
Age of the child	Two years and seven months
Onset & duration of diarrhea	Before 12 h
Frequency of stools	5 times
Presence of fever	No
Presence of blood and/or mucus in stools	No

**Table 2:** Socio-demographics/sample characteristics (n = 303).

*Variable*	*Options*	*Frequency* (*n*)	*%*
Gender	Female	155	51.2
	Male	148	48.8
Age Group	20–30	123	40.6
	31–40	76	25.1
	41–50	95	31.4
	> 50	9	3
Exact Age	34.59 (SD ± 8.07)		
Job Profile	Pharmacy Technician	76	25.1
	Staff Pharmacists	203	67
	Pharmacy Manager	24	7.9
Educational Qualification	Bachelors	46	15.2
	Pharm. D	252	83.2
	Masters	5	1.7
Experience (*In Years*)	<5 Years	79	26.1
	5–10 Years	188	62
	>10 Years	36	11.9

A relatively similar completion time (i.e. six minutes) as the pilot study was also noticed in the main study. The questionnaire was distributed to around 350 community pharmacists, out of which 303 consented and completed the questionnaire. The response rate was observed as 86.28 %.

### 3.2 Reliability analysis

One standard statistic was used to assess the reliability of the instrument. The pilot study sample was also used to test the reliability and the internal consistency of the instrument. Cronbach’s alpha was used to assess the internal consistency of the different items of the scale. The alpha coefficients for all the items of the survey instrument were noted to be greater than (or) equal to the 0.70 threshold for almost all sections except for the participants’ practices towards drug recommendations which had (α = 0.67) (see Table 3), indicating good reliability for the developed scale (Fornell and Larcker, 1981, Fauville et al., 2021, Alqahtani et al., 2022). The pilot study sample was subsequently excluded from the final analysis.

**Table 3:** Internal consistency of the questionnaire.

Section	Cronbach’s Alpha	
	*Self-report survey*	*Simulated Patient Visits*
Participants’ knowledge towards history taking of child suffering from Acute childhood diarrhea	0.86	0.92
Participants’ knowledge towards drug recommendations for Acute childhood diarrhea	0.73	0.67
Participants’ Attitude towards information on food & fluid intake for patients suffering from Acute childhood diarrhea	0.78	0.79
Participants’ Practices towards Management of Acute childhood diarrhea	0.70	0.73

### 3.3 Participants’ knowledge and practices towards patients’ history taking

Table 4, elucidates the participants’ responses to questions assessing their knowledge (self-report survey) as well as their practices (simulated patient visits) towards history taking of children suffering from acute diarrhea. As part of history taking, almost all of the participants (93.1 %) in the self-report survey inquired about the symptoms of dehydration as chief complaint. Whereas, in actual practice this percentage was only 69 %, yielding a statistically significant finding (*p <* 0.01). Child’s age was inquired by majority of participants in both the self-report survey (92.1 %) and during the simulated patient visits (84 %), with the finding being statistically significant (*p <* 0.001). Similarly high percentages of participants inquired about the duration of diarrheal episodes (83.8 %), frequency of diarrheal episodes (76.2 %) and the presence of fever (78.5 %) as part of history taking in the self-report survey. On the contrary, in actual practice the duration of diarrheal episodes (31 %), frequency of diarrheal episodes (63 %) and the presence of fever (47 %) were less frequently inquired. Inquiring about the duration of the diarrheal episodes (*p <* 0.05), frequency of diarrheal episodes (*p <* 0.001) were also statistically significant, while inquiring about the presence of fever did not have any statistical significance (*p >* 0.05). The presence of mucus (58.1 % vs 39 %) and the presence of blood in stools (62.7 % vs 30 %) were the least frequently inquired as part of history taking in both the self-report survey and the simulated patient visits respectively and these findings were also statistically significant (*p <* 0.01; *p <* 0.001).

**Table 4:** Participants’ knowledge and practices towards patient’s history taking.

	***Knowledge***		***Practices***		*p-value*
*Questions*	*Self-report survey*		*Simulated patient visits*		
	*Yes ‘n (%)’*	*No ‘n (%)’*	*Yes ‘n (%)’*	*No ‘n (%)’*	
Symptoms of dehydration as chief complaint	282 (93.1)	21 (6.9)	69 (69)	31 (31)	0.003
Child’s age	279 (92.1)	24 (7.9)	84 (84)	16 (16)	*p<*0.001
Child’s weight	203 (67)	100 (33)	44 (44)	56 (56)	*p<*0.001
Child’s medication history	223 (73.6)	80 (26.4)	35 (35)	65 (65)	0.001
Duration of diarrheal episodes	254 (83.8)	49 (16.2)	31 (31)	69 (69)	0.02
Frequency of diarrheal episodes	231 (76.2)	72 (23.8)	63 (63)	37 (37)	*p<*0.001
Presence of mucus in the stools	176 (58.1)	127 (41.9)	39 (39)	61 (61)	0.001
Presence of blood in the stools	190 (62.7)	113 (37.3)	30 (30)	70 (70)	*p<*0.001
Presence of fever	238 (78.5)	65 (21.5)	47 (47)	53 (53)	0.06

### 3.4 Participants’ knowledge and practices towards drug recommendations

Table 5, shows the study participants’ responses towards the drug recommendations for the management of acute childhood diarrhea. It was noticed that most of the participants in the self-report survey and in the simulated patient visits (88.1 % vs 90 %) respectively recommended the use ORS to their patients, yielding a statistically significant finding (*p <* 0.001). Only around half of the sample in both the studies (57.8 % vs 59) recommended zinc supplements along with ORS. Similarly, just over half of participants (53.8 % vs 61 %) in both the studies (self-report survey and simulated patient visits) did not recommend the use of antispasmodics to their patients, resulting in statistically significant finding (*p <* 0.001). Less than half of participants in the self-report survey (45.2 %) and the simulated patient visits (43 %) recommended the use of other agents (adsorbents like Kaolin) for the management of acute childhood diarrhea. This finding was also statistically significant (*p <* 0.001).

**Table 5:** Participants’ knowledge and practices towards drug recommendations.

*Questions*	*Self-report survey*		*Simulated patient visits*		*p-value*
	*Yes* *‘n* (*%*)*’*	*No* *‘n* (*%*)*’*	*Yes* *‘n* (*%*)*’*	*No* *‘n* (*%*)*’*	
1. ORS is given to alleviate diarrheal symptoms?	267 (88.1)	36 (11.9)	90 (90)	10 (10)	*p <* 0.001
2. ORS + Zinc supplements are given to alleviate diarrheal symptoms?	175 (57.8)	128 (42.2)	37 (37)	63 (63)	*p <* 0.001
3. ORS + Antimicrobials are given to alleviate diarrheal symptoms?	270 (89.1)	33 (10.9)	78 (78)	22 (22)	0.46
4. Antispasmodics are given to alleviate diarrheal symptoms?	140 (46.2)	163 (53.8)	35 (35)	65 (65)	*p <* 0.001
5. Other medications (Kaolin, etc.) are given to alleviate diarrheal symptoms?	137 (45.2)	166 (54.8)	43 (43)	57 (57)	*p <* 0.001

### 3.5 Participants’ attitude and practices towards instructions for food & fluid intake

Table 6, elucidates the results of participants’ responses from the self-report survey to questions assessing their attitudes towards instructions for food & fluid intake. With regards to the statement *“Do you advice to continue normal feeding and increasing the fluid intake during diarrhea”*, it was noticed that more than three quarters of the sample (75.9 %) strongly agreed with this statement showing an excellent attitude. On the other hand, in actual practice only 27 % of the participants gave advice to continue normal feeding and to increase fluid intake during diarrhea thus exhibiting poor practice, this finding did not have any statistical significance (*p >* 0.05). (Table 6). Poor attitude was also noticed when more than half (52.5 %) of the sample either agreed (21.5 %) or strongly agreed (31 %) to the statement *‘No need to advice about food and fluid during diarrhea illness’* in the self-report survey (Table 6).

**Table 6:** Participants’ attitude and practices towards instructions for food & fluid intake.

Self-report survey				
*Questions*	Strongly Disagree 1	Disagree 2	Agree 3	Strongly Agree 4
1. Advice to continue normal feeding and increasing the fluid intake during diarrhea?	39 (12.9 %)	16 (5.3 %)	18 (5.9 %)	230 (75.9 %)
	Strongly Disagree 4	Disagree 3	Agree 2	Strongly Agree 1
*2. Advice to withhold breastfeeding during diarrhea?	119 (39.3 %)	15 (5 %)	138 (45.5 %)	31 (10.2)
*3. Advice only to increase fluid intake during their child’s diarrhea?	88 (29 %)	36 (11.9 %)	125 (41.3 %)	54 (17.8 %)
*4. Advice only to continue feeding during their child’s diarrhea?	142 (46.9 %)	14 (4.6 %)	100 (33 %)	47 (15.5 %)
*5. No need to advice about food and fluid during diarrhea illness?	108 (35.6 %)	36 (11.9 %)	65 (21.5 %)	94 (31 %)
*Items 2, 3, 4, 5 are negative statements and hence are reverse coded 1 - Strongly Agree; 2 - Agree; 3 - Disagree; 4 - Strongly Disagree*				

Participants’ practices towards instructions for food & fluid intake - *Simulated patient visits*				

		*Yes ‘n* (*%*)*’*	*No ‘n* (*%*)*’*	*p-value*

1. Advice to continue the normal feeding and increasing the fluid intake during diarrhea?		27 (27 %)	73 (73 %)	0.07
*2. Advice to withhold breastfeeding during diarrhea?		0 (0 %)	100 (100 %)	–
*3. Advice only to increase fluid intake during their child’s diarrhea?		0 (0 %)	100 (100 %)	–
*4. Advice only to continue feeding during their child’s diarrhea?		0 (0 %)	100 (100 %)	–
*5. No need to advice about food and fluid during diarrhea illness?		0 (0 %)	100 (100 %)	–

### 3.6 Participants’ practices towards ORS preparation & storage

Table 7, illustrates the participants’ responses to questions assessing their practices towards instructions provided to the patients’ parents regarding preparation, storage and how and when to give ORS. More than half of participants (69 %) of the self-report survey responded that they instruct the child’s parents on how to prepare ORS. But, during the simulated patient visit it was noted that more than half of the participants (56 %) did not provide any such instructions. This finding did not have any statistical significance (*p >* 0.05). Similar poor practices were also seen during simulated patient visits when a high percentage of participants (71 %) did not provide any instructions on the storage of ORS after reconstitution, yielding a statistically significant finding (*p <* 0.05). Similarly high proportion (83 %) of sample did not provide any instructions on how and when to give their child ORS. This finding also did not have any statistical significance (*p >* 0.05).

**Table 7:** Participants’ practices towards instructions for ORS preparation & storage.

	***Self-report survey***		***Simulated patient visit***		*p-value*
	*Yes ‘n* (*%*)*’*	*No ‘n* (*%*)*’*	*Yes ‘n* (*%*)*’*	*No ‘n* (*%*)*’*	
1. Do you show the child’s parents on how to prepare ORS?	209 (69 %)	94 (31 %)	44 (44 %)	56 (56%)	0.23
2. Do you show the child’s parents on how to store ORS after reconstitution?	205 (67.7 %)	98 (32.3 %)	29 (29 %)	71 (71%)	0.03
3. Do you instruct the child’s parents on how and when to give their child ORS?	151 (49.8 %)	152 (50.2 %)	17 (17%)	83 (83% )	0.08

### 3.7 Association between participants’ socio-demographics and different categories of knowledge of patients’ history taking

Table 8, depicts the association between participants’ socio-demographics and different categories of knowledge. The participants’ knowledge of patient’s history taking was significantly associated with having Pharm.D degree (*χ^2^ =* 14.17; *p =* 0.003), being a staff pharmacists or a pharmacy manager (*χ^2^ =* 12.67*; p =* 0.012), being above the age of 30 years (*χ^2^ =* 152.40*; p <* 0.001), and having experience greater than five years (*χ^2^ =* 104.90*; p <* 0.001) Participants’ gender was not significantly associated with their knowledge of history taking.

**Table 8:** Crosstabulations between participants’ demographics and different categories of knowledge of patients’ history taking.

	*Poor Knowledge*	*Moderate Knowledge*	*Excellent Knowledge*	*Total* *n* (*%*)	(*Chi square*) *χ^2^*	*p-value*
***Educational Qualification***						
Bachelors	20 (27.4 %)	10 (16.4 %)	16 (9.5 %)	46 (15.2 %)	*14.17*	*p =* 0.003
Pharm. D	53 (72.6 %)	51 (83.6 %)	148 (87.6 %)	252 (83.2 %)		
Masters	0 (0.0 %)	0 (0.0 %)	5 (3.0 %)	5 (1.7 %)		
***Gender***						
Female	33 (45.2 %)	31 (50.8 %)	91 (53.8 %)	155 (51.2 %)	*1.52*	*p = 0.47*
Male	40 (54.8 %)	30 (49.2 %)	78 (46.2 %)	148 (48.8 %)		
***Job Profile***						
Pharmacy Technician	29 (39.7 %)	14 (23.0 %)	33 (19.5 %)	76 (25.1 %)	*12.67*	*p =* 0.012
Staff Pharmacists	41 (56.2 %)	44 (72.1 %)	118 (69.8 %)	203 (67.0 %)		
Pharmacy Manager	3 (4.1 %)	3 (4.9 %)	18 (10.7 %)	24 (7.9 %)		
***Age Group***						
21–30	64 (87.7 %)	39 (63.9 %)	20 (11.8 %)	123 (40.6 %)	*152.40*	*p <* 0.001
31–40	6 (8.2 %)	13 (21.3 %)	57 (33.7 %)	76 (25.1 %)		
41–50	3 (4.1 %)	9 (14.8 %)	83 (49.1 %)	95 (31.4 %)		
> 51	0 (0.0 %)	0 (0.0 %)	9 (5.3 %)	9 (3.0 %)		
***Experience* (*in years*)**						
< 5	50 (68.5 %)	15 (24.6 %)	14 (8.3 %)	79 (26.1 %)	*104.90*	*p <* 0.001
5–10	23 (31.5 %)	43 (70.5 %)	122 (72.2 %)	188 (62.0 %)		
> 10	0 (0.0 %)	3 (4.9 %)	33 (19.5 %)	36 (11.9 %)		

### 3.8 Association between participants’ knowledge of patient’s history taking and attitude towards instructions for food & fluid intake

Table 9, shows the results of crosstabulations between different categories of knowledge of patient’s history taking and attitude towards instructions for food & fluid intake from the self-report survey. It was noticed that 67 % of participants with excellent knowledge of history taking of patients with acute childhood diarrhea also demonstrated excellent attitudes towards instructions being given to patients about food & fluid intake, and correspondingly yielding a statistically significant association between the two variables. (*χ^2^* = 26.99; *p <* 0.001).

**Table 9:** Crosstabulations of different categories of Knowledge of patient’s history taking and attitude towards instructions for food & fluid intake.

	*Poor Attitude*	*Moderate Attitude*	*Excellent Attitude*	*Total* *n* (*%*)	(*Chi square*) *χ^2^*	*p-value*
Poor Knowledge	27 (45.8 %)	26 (20.2 %)	20 (17.4 %)	73 (24.1 %)	26.99	*p <* 0.001
Moderate Knowledge	15 (25.4 %)	28 (21.7 %)	18 (15.7 %)	61 (20.1 %)		
Excellent Knowledge	17 (28.8 %)	75 (58.1 %)	77 (67.0 %)	169 (55.8 %)		

### 3.9 Association between participants’ attitude towards instructions for food & fluid intake and practices for ORS preparation & storage

Table 10, elucidates the results of crosstabulations between different categories of attitude towards instructions for food & fluid intake and practices of providing information for ORS preparation & storage. It was noticed that a very high percentage of the sample (69.8 %) with excellent attitude towards instructions being given to patients about food & fluid intake also displayed excellent practices towards the instructions being provided to the patients for ORS preparation and storage, and thus yielding a statistically significant association between the variables (*χ^2^* = 99.39; *p <* 0.001).

**Table 10:** Crosstabulations of different categories of attitude towards instructions for food & fluid intake and practices of providing information for ORS preparation & storage (Survey study).

	*Poor Practices*	*Moderate Practices*	*Excellent Practices*	*Total* *n* (*%*)	(*Chi square*) *χ^2^*	*p-value*
Poor Attitude	38 (39.2 %)	14 (15.6 %)	7 (6.0 %)	59 (19.5 %)	99.39	*p <* 0.001
Moderate Attitude	46 (47.4 %)	55 (61.1 %)	28 (24.1 %)	129 (42.6 %)		
Excellent Attitude	13 (13.4 %)	21 (23.3 %)	81 (69.8 %)	115 (38.0 %)		

### 3.10 Correlation between knowledge of patient’s history taking score, attitudes towards instructions for food & fluid intake score and their corresponding practice scores

Pearson’s correlation coefficient *‘r’* was calculated for the scores of different sections. The knowledge of patient’s history taking and attitudes towards instructions for food & fluid intake scores were taken from the self-report survey and compared with their corresponding practice scores from the simulated patient visits. Significant positive correlations were noticed between participants’ knowledge score about history taking of the patients with acute childhood diarrhea and the practice of patient’s history taking score of the same (r = 0.65; *p < 0.01*)*.* Similar significant positive correlations were also noticed between participants’ knowledge score regarding drugs recommended for the management of acute childhood diarrhea and their corresponding practice score (r = 0.71; *p < 0.01*)*.* Participants’ attitude towards instructions for food & fluid intake score was also significantly and positively correlated with its corresponding practice score (r = 0.44; *p < 0.05*) (Table 11).

**Table 11:** Correlation between different scores.

	*Pearson’s Correlation Coefficient ‘r’*	*p-value*
Knowledge of patient’s history taking Score vs Practice of patient’s history taking Score	0.65	*p < 0.01*
Knowledge of drug recommendations Score vs Practice of drug recommendations Score	0.71	*p < 0.01*
Attitudes towards instructions for food & fluid intake Score vs Practice towards instructions for food & fluid intake Score	0.44	*p < 0.05*

*Knowledge Score was determined from the self-report survey; Practice score was determined from the simulated patient visits.

## 4 Discussion

The present study explored community pharmacy professionals’ differential knowledge and attitude towards the management of acute childhood diarrhea through a self-report survey and compared them with their corresponding practices as assessed through simulated patient visits. Currently, throughout the world the simulated patient visits are being regarded as the best and the simplest method to assess the dispensing practices of the community pharmacists (Diwan et al., 2015, Watson et al., 2004). And hence, this method could as well be regarded as the best approach to ascertain the practices of community pharmacy professionals in combating diarrhea and its associated problems. The present study pivots on a community pharmacy professionals’ knowledge towards history taking, recommending drugs, their attitudes towards appropriate food and fluid intake, as part of the management of acute childhood diarrhea. According to the WHO, recommendations such as the recurrence and continuance of diarrhea, past and present illnesses, blood in stools, vomiting episodes, vaccination, type of food and fluid intake, and the medications need to be followed (WHO, 1987). The WHO design to control diarrhea mandates assessment of the onset along with the duration of diarrhea. Nine key components relatively similar to that of the 11 used by Mengistu et al. (2019), were analyzed to estimate the knowledge of community pharmacy professionals towards history taking of the patients suffering from acute childhood diarrhea. Patient’s age (92.1 % vs 84 %) was the most commonly taken history in both the self-report survey and during the simulated patient visits, thus yielding a statistically significant finding (*p <* 0.001). A similar observation was also made by Mengistu et al. (2019), who conducted a study in Ethiopia, to assess the involvement of community pharmacy professionals in the management of acute childhood diarrhea, and reported that a very high proportion (98.1 %) of their sample inquired about the patient’s age as part of history taking in both the self-report survey as well as during the simulated patient visits, but the level of significance of their results were not reported by Mengistu et al. (2019). Ogbo et al. (2014), also made a statistically significant (*p <* 0.0001) observation wherein they found 76.7 % of participants of the questionnaire study used child’s age as an evaluation indicator. Sancar et al. (2015), also made a comparable statistically significant observation (*p <* 0.001), wherein they noticed a very high percentage (94 %) of participants inquired about the patient’s age. Even though a relatively analogous observation was also made by Hussain & Ibrahim (2012), who reported that 83.3 % of their sample inquired about the patients’ age as part of history taking, but the level of significance was not reported for this variable by Hussain & Ibrahim (2012).

Although, symptoms of dehydration (93.1 % vs 81.9 %), duration of diarrheal episodes (83.8 % vs 96.2 %), frequency of diarrheal episodes (76.2 % vs 97.1 %), and presence of fever (78.5 % vs 89.5 %) were some of the other important components of history taking by participants in the self-report survey of the present study and by Mengistu et al. (2019), but unfortunately in actual practice (i.e. during the simulated patient visits) symptoms of dehydration (69 % vs 0 %), duration of diarrheal episodes (31 % vs 20 %), frequency of diarrheal episodes (63 % vs 24.8 %), presence of fever (47 % vs 24.8 %) and taking patients’ medication history (35 % vs 0 %) were not inquired as frequently as part of history taking in the present study as well as by Mengistu et al. (2019). Mengistu et al. (2019), did not report the level of significance for their results. In the present study inquiring about the duration of diarrheal episodes (*p <* 0.05), frequency of diarrheal episodes (*p <* 0.001), presence of mucus in stools (*p <* 0.01), and the presence of blood in stools (*p <* 0.001) were all found to be statistically significant. These findings are comparable with Ogbo et al. (2014), who also made similar statistically significant (*p <* 0.0001) observations wherein they found that 76.7 % of their self-report survey sample used frequency of stools, presence of mucus in stools, presence of blood in stools as evaluation indicators before recommending drugs. With regards to medication history taking, while the self-report survey showed that high percentage of participants (73.6 % vs 81.9 %) inquired about their patients’ medication history this wasn’t seen during the simulated patient visits, as only (35 % vs 0 %) were seen practicing this, both in the present study and in the study conducted by Mengistu et al. (2019). A similar finding was also reported by a Turkish study where participants did not collect patients’ medication history during simulated patient visits (Sancar et al., 2015). Hussain & Ibrahim (2012), also reported a relatively similar finding wherein a very low percentage of their study participants (7.8 %) took their patients’ medication history. Thus, highlighting poor history taking in actual practice. Level of significance for this variable was not reported in either of the studies.

In the self-report survey, questions regarding the presence of blood and mucus in stool accompanied by fever provided insight into the intensity of infection. Just over half of the sample studied inquired about blood in the stools (62.7 %) and mucus in stools (58.1 %) in the self-report survey, this percentage drastically came down in the simulated patient visits to 30 % and 39 % for the presence of blood and mucus in stools respectively, showing poor practices. Similar poor dispensing practices were also observed by Mengistu et al. (2019), who reported a very low percentage (43.8 %) of their sample to inquire about the presence of mucus or blood in stools during simulated patient visits. This discrepancy between the reported rates in the self-report survey and the simulated patient visits highlights poor practices. This information about the severity of the infection could be helpful to the community pharmacy professionals in identifying the possible causes of acute childhood diarrhea, and then to either recommend further diagnosis, (or) to provide guidance on management strategies with regular follow-ups, and in some cases initiate proper referrals for care and hospitalization (Mengistu et al., 2019).

The present framework requires a community pharmacist to have in-depth knowledge and proficiency in recommending appropriate OTC medication which could go a long way in the improvement of public health and safe use of medicines (Mengistu et al., 2019). The use of ORS was the most common recommendation in both the self-report survey (88.1 %) and during the simulated patient visits (90 %), thus resulting in a statistically significant finding (*p <* 0.001). Relatively similar statistically significant finding (*p <* 0.0001) was also reported by Ogbo et al. who conducted a study in Nigeria, and found that 62.6 % of the respondents also recommended the use of ORT (Ogbo et al., 2014). Even though, similar pattern was seen for the recommendation of the combination of ORS and antimicrobials as seen from the self-report survey (89.1 %) and simulated patient visits’ data (78 %), these findings were not statistically significant (*p >* 0.05). These findings are very similar to Mengistu et al. (2019), who also reported a very high proportion of their sample to recommend antimicrobials during simulated patient visits (86.7 %) and in the self-report survey (50.5 %). Although these findings are somewhat similar to Ogbo et al. (2014), who also reported more than half of the sample (62.4 %) of the simulation visits to have recommended ORT along with antibiotics, they differ from the findings of the present study in that Ogbo et al. (2014) reported these findings to be statistically significant (*p <* 0.0001) where as we reported them not to have any statistical significance (*p >* 0.05). Our findings are in contrast with Wongsaroj et al. (1997), who conducted a study in Thailand to study the management of acute childhood diarrhea and reported a very low percentage of their sample (25.6 %) to recommend ORS along with antibiotics. Sancar et al. (2015), also reported that only 0.01 % of their study participants recommended antibiotics for the management of diarrhea.

Dehydration-related nutritional deficiency can be minimized with obligatory fluid replacement, followed by the possible use of zinc supplements to reduce the duration and severity of diarrhea (Hussain & Ibrahim, 2012). Although, more than half of participants (57.8 %) in the self-report survey recommended ORS along with zinc supplements, but in actual practice only (37 %) recommended the above combination thus, highlighting poor practices. Antispasmodics and other drugs (e.g. Kaolin, etc) were the least recommended drugs both in the self-report survey (46.2 % vs 45.2 %) and during the simulated patient visits (35 % vs 43 %) respectively. These findings were statistically significant (*p <* 0.001). Ogbo et al. (2014), also reported a comparable statistically significant observation (*p <* 0.0001) when reporting kaolin and antispasmodics as the least recommended combination (13.1 % vs 22.6 %) in questionnaire study as well as during the simulation patient visits. These findings are relatively similar to that of Hussain & Ibrahim (2012), who also reported only 17 % of their sample to recommend other drugs (e.g. Kaolin, pectin, etc.). However, the level of significance was not reported for this variable by Hussain & Ibrahim (2012).

WHO, gives prominence to the diet suggested to the patients of acute diarrhea during and after the illness (WHO, 1994, WHO, 1994). To avoid dehydration, malnutrition, and fluid imbalance, continuous feeding and fluid intake are essential. In the present study, more than three quarters (75.9 %) of the sample in the self-report survey advised to continue normal feeding and to increase fluid intake, thus highlighting excellent attitude, where as in actual practice only 27 % of the sample advised their patients about continuing normal feeding and increasing fluid intake. This variation in the reported rates of the self-report survey and simulated patient visits highlights poor practices towards instructions on food and fluid intake.

With regards to the information on the preparation and storage of ORS, it was observed that even though high proportion of the sample in the self-report survey reported giving instructions on how to prepare the ORS (69 %) and how to store the ORS (67.7 %) but during the simulated patient visits only 44 % of the sample actually provided information on how to prepare the ORS and 29 % on how to store the ORS. This discrepancy between the reported rates of self-report survey and the simulated patient visit also highlights wide-spread poor practices towards the instructions being given on how to prepare and store ORS in the current sample. Of these findings, only the instructions on how to properly store ORS was found to be statistically significant (*p <* 0.05). This finding draws relative comparison with Sancar et al. (2015), who also reported a statistically significant observation (*p <* 0.001) for the instructions on how to properly store medications used in the treatment of diarrhea.

Statistically significant associations were seen between participants’ knowledge of history taking and their educational qualification (*p =* 0.003), job profile (*p =* 0.012), age group (*p <* 0.001), and experience (*p <* 0.001). Participants’ gender was not found to be significantly associated with their knowledge of history taking. In terms of age, these findings are in contrast with Siltrakool et al. (2021), who reported that the community pharmacists’ age had no association with their knowledge, attitudes and practices. The above study also reported that Pharm.D qualification was associated with better knowledge. This finding is consistent with our study wherein we also observed that 87.6 % of participants with excellent knowledge had Pharm.D qualification (*p <* 0.01). Siltrakool et al. (2021), also reported the community pharmacists’ experience to have varying affect on their knowledge, attitudes and practices. We also made a comparable observation wherein we found that the community pharmacy professionals’ experience had a significant association with their knowledge (*p <* 0.001). Significant positive correlations were also seen between the participants’ knowledge score (self-report survey) and practice score (simulated patient visits) regarding patients’ history taking (r = 0.65; *p < 0.01*), drug recommendations for the management of acute childhood diarrhea (r = 0.71; *p < 0.01*)*,* and in providing information regarding food and fluid intake (r = 0.44; *p < 0.05*)*.* These aforementioned findings demonstrate that as the score of one of the variable increases so does the score of the other (Alnohair et al., 2021, Syed et al., 2022). From these findings we can also deduce that the three KAP components (i.e., knowledge, attitude and practice) are correlated. Yousaf et al. (2020), also reported the knowledge, attitude and practice scores to be correlated with each other. A recently conducted study amongst community pharmacists in Lebanon, also reported significant positive correlations between the three KAP components. They made a conclusion that an increase in one of the KAP component directly leads to an increase of the other KAP components and vice-versa (Hallit et al., 2020). Mehralian et al. (2014), also showed that the community pharmacists’ knowledge significantly affected their attitudes as well as their practices. We were also able to make comparatively similar observations in the present study wherein, it was reported that a high percentage of study participants (67 %) with excellent knowledge also showed excellent attitude towards the management of acute childhood diarrhea (*p* < 0.001). A similar observation was also made between the participant’s attitude and practice wherein a high proportion of the sample exhibiting excellent attitudes (69.8 %) also demonstrated excellent practices towards the management of acute childhood diarrhea (*p* < 0.001). Hajj et al. (2019), also made a similar observation where they reported community pharmacists’ better knowledge was significantly associated with better attitude and better practice.

## 5 Strengths and limitations

To the best of the author’s knowledge this is the first study in Jazan province to make use of self-report survey to assess the participant’s knowledge and attitude and to compare that with their corresponding practices through simulated patient visits amongst community pharmacy professionals towards the management of acute childhood diarrhea.

Simulated patient visits have not extensively been used for data collection in Saudi Arabia and especially in Jazan province, so this new technique might have been prone to some limitations. One major limitation of the present study is its inability to conduct simulated patient visits across all community pharmacies involved in the self-report survey. As simulation patient visits are an arduous process, completely dependent upon the simulation patients’ ability to recall their training, sometimes they might also forget to ask questions important for achieving the study outcomes and after the completed visits, the simulated patients are required to transcribe the obtained information into data collection sheets and hence owing to these reasons are subject to recall bias (Firstly the training, then the questions and lastly the responses provided) and it proves to be a major limitation of simulated patient visits. Due to these aforementioned reasons and due to the fact that we only had three volunteers as simulated patients along with their relative inexperience the interns who volunteered as simulated cases only consented to 100 such visits. Moreover, the training received by the simulated cases depended solely upon experience of the clinical pharmacist training them. Lack of training, experience, and expertise of the trainer would definitely impact the quality of the collected data. Additionally, the presence of more than one pharmacist might have elicited different responses. Some other potential limitations include the cross-sectional design not making it possible for the assessment of causality. The study was conducted only amongst the community pharmacy professionals, hospital and clinical pharmacists were not included. Moreover, the present study was conducted in a single province of Saudi Arabia (i.e., Jazan) and hence, we cannot claim that the results can be generalized to other parts of the country. The study participants may not have been representative owing to convenience sampling. Furthermore, a self-report survey is always prone to potential recall bias and the convenience sampling technique might have led to possibility of selection bias.

## 6 Conclusion

In the present study, even though we observed improved performance in collecting patients’ history, drug recommendations, and instructions for food and fluid intake and in providing instructions for the preparation and the storage of ORS in the self report survey the performance level of the sample greatly fell in actual practice as assessed through simulated patient visits. This discrepancy between the reported rates highlights wide-spread poor practices towards the management of acute childhood diarrhea in the current sample. This warrants for the need of educational initiatives and training programs for the community pharmacy professionals to highlight the need and to improve their actual dispensing practices. The findings of the present study have also shown that simulated patient visits are a simple and a robust method of assessing the actual dispensing practices of community pharmacy professionals.

## Future prospective

A similar study should be conducted at the national level and all the community pharmacies where data was collected using a self-report survey should be included in simulated patient visits.

## Author contributions

EMA designed the original project and drafted the proposal. YAMM, MAIA, BAH, MHA, RMS, AK, RT collected the data. NKS, EMA analyzed the data and wrote the manuscript. NKS, SSA, AK & OB revised the manuscript. All authors read and approved the final version of the manuscript.

## Funding

The authors did not receive any funding for conducting the present study.

## Declaration of Competing Interest

The authors declare that they have no known competing financial interests or personal relationships that could have appeared to influence the work reported in this paper.
