# Supplementary material for: Self-reported and the actual involvement of community pharmacy professionals in the management of acute childhood diarrhea in Jazan Province, Saudi Arabia: Simulated patient visits and survey study
Source: Saudi Pharm J. 2022 Jul 29;30(10):1497–506. doi: 10.1016/j.jsps.2022.07.012 (PMC9649352; doi:10.1016/j.jsps.2022.07.012)
Supplement: Supplementary data 1 [file mmc1.docx]

**APPENDIX - 1: STUDY QUESTIONNAIRE**

**GENERAL INSTRUCTIONS & INFORMED CONSENT:**

1. **The Questionnaire contains TWO sections,**
2. **Section - 2, is further subdivided into 2 sections (2.1 – 2.4)**
3. **Kindly answer all the questions HONESTLY.**
4. **Kindly tick the most appropriate answer. Use legible ink & do not over-write.**
5. **The data generated from this study would only be used for scientific purposes. No personal information is being sought. Confidentiality of the data is paramount to us.**
6. **The participants can partake in this study by putting their initials to the consent form.**
7. **The participants can withdraw their consent at any point of time.**
8. **Should there be any doubts feel free to contact the PRINCIPAL INVESTIGATOR Dr. Nabeel Kashan Syed (**[**nsyed@jazanu.edu.sa**](mailto:nsyed@jazanu.edu.sa)**).**

| **INFORMED CONSENT:**  **This Questionnaire is only for student research and will only be used for scientific purpose. No personal information provided will be used.**  **I have not been forced to be part of this survey. I am participating in this survey by answering this questionnaire and giving my consent out of my own free will.**  **Signature…………………………………………………… Date: ……………** |
| --- |

| **SECTION - 1: DEMOGRAPHICS** | | | |
| --- | --- | --- | --- |
| **S.No** | **CATEGORY** | **OPTIONS** | **TICK HERE** |
| **D1** | **GENDER:** | 1: Male | **🞏** |
|  |  | 2. Female | **🞏** |
|  | | | |
| **D2** | **AGE GROUP**  *(in years)* | 1: 21 - 30 | **🞏** |
|  |  | 2: 31 - 40 | **🞏** |
|  |  | 3: 41 - 50 | **🞏** |
|  |  | 4: > 50 | **🞏** |
|  | | | |
| **D3** | **QUALIFICATION** | 1: Diploma | **🞏** |
|  |  | 2: Bachelors in Pharmacy | **🞏** |
|  |  | 3: Pharm.D | **🞏** |
|  |  | 4: Masters | **🞏** |
|  | | | |
| **D4** | **JOB PROFILE** | 1: Pharmacy Technician | **🞏** |
|  |  | 2: Staff Pharmacists | **🞏** |
|  |  | 3: Pharmacy Manager | **🞏** |
|  | | | |
| **D5** | **EXPERIENCE**  *(in years)* | 1: < 5 | **🞏** |
|  |  | 2: 5 - 10 | **🞏** |
|  |  | 3: > 10 | **🞏** |
|  | | | |

| **SECTION - 2.1: PARTICIPANTS’ KNOWLEDGE TOWARDS PATIENTS’ HISTORY TAKING** | | | | | |
| --- | --- | --- | --- | --- | --- |
| S.No | Question | Options | | | |
| Which of the following are important components of history taking for acute childhood diarrhea? | | | | | |
| 1 | Symptoms of dehydration as chief complain | 1:Yes | **🞏** | 0:No | **🞏** |
| 2 | Patient’s age | 1:Yes | **🞏** | 0:No | **🞏** |
| 3 | Patient’s weight | 1:Yes | **🞏** | 0:No | **🞏** |
| 4 | Patient’s medication history | 1:Yes | **🞏** | 0:No | **🞏** |
| 5 | Duration of diarrheal episodes | 1:Yes | **🞏** | 0:No | **🞏** |
| 6 | Frequency of diarrheal episodes | 1:Yes | **🞏** | 0:No | **🞏** |
| 7 | Presence of mucus in the stools | 1:Yes | **🞏** | 0:No | **🞏** |
| 8 | Presence of blood in the stools | 1:Yes | **🞏** | 0:No | **🞏** |
| 9 | Presence of fever | 1:Yes | **🞏** | 0:No | **🞏** |

| **SECTION - 2.2: PARTICIPANTS’ KNOWLEDGE TOWARDS RECOMMENDATIONS FOR THE MANAGEMENT OF CHILDHOOD DIARRHEA** | | | | | |
| --- | --- | --- | --- | --- | --- |
| S.No | Question | Options | | | |
| 1 | ORS is given to alleviate diarrheal symptoms? | 1:Yes | **🞏** | 0:No | **🞏** |
| 2 | ORS + Zinc supplements are given to alleviate diarrheal symptoms? | 1:Yes | **🞏** | 0:No | **🞏** |
| 3 | ORS + Antimicrobials are given to alleviate diarrheal symptoms? | 1:Yes | **🞏** | 0:No | **🞏** |
| 4 | Antispasmodics are given to alleviate diarrheal symptoms? | 1:Yes | **🞏** | 0:No | **🞏** |
| 5 | Other medications (Kaolin, etc.) are given to alleviate diarrheal symptoms? | 1:Yes | **🞏** | 0:No | **🞏** |

| **SECTION - 2.3: PARTICIPANTS’ ATTITUDE TOWARDS FOOD & FLUID INTAKE** | | | | | |
| --- | --- | --- | --- | --- | --- |
| S.No | Question | Options | | | |
|  |  | **Strongly**  **Disagree**  **1** | **Disagree**  **2** | **Agree**  **3** | **Strongly**  **Agree**  **4** |
| 1 | Advice to continue the normal feeding and increasing the fluid intake during diarrhea? | **🞏** | **🞏** | **🞏** | **🞏** |
| 2 | *Advice to withhold breastfeeding during diarrhea? | **🞏** | **🞏** | **🞏** | **🞏** |
| 3 | *Advice only to increase fluid intake during their child’s diarrhea? | **🞏** | **🞏** | **🞏** | **🞏** |
| 4 | *Advice only to continue feeding during their child’s diarrhea? | **🞏** | **🞏** | **🞏** | **🞏** |
| 5 | * No need to advice about food and fluid during diarrhea illness? | **🞏** | **🞏** | **🞏** | **🞏** |
| Items 2, 3, 4, 5 are reverse coded 1 - Strongly Agree 2 - Agree; 3 - Disagree; 4 - Strongly Disagree | | | | | |

| **SECTION - 2.4: PARTICIPANTS’ PRACTICES TOWARDS PREPARATION & STORAGE OF ORS** | | | | | |
| --- | --- | --- | --- | --- | --- |
| S.No | Question | Options | | | |
| 1 | Do you show the child’s parents on how to prepare ORS? | 1:Yes | **🞏** | 0:No | **🞏** |
| 2 | Do you show the child’s parents on how to store ORS after reconstitution? | 1:Yes | **🞏** | 0:No | **🞏** |
| 3 | Do you instruct the child’s parents on how and when to give their child ORS? | 1:Yes | **🞏** | 0:No | **🞏** |
|  | | | | | |

**THANK YOU VERY MUCH INDEED FOR YOUR TIME & PATIENCE**
